# Exploring GWAS and genomic prediction to improve Septoria tritici blotch resistance in wheat

**DOI:** 10.1038/s41598-023-42856-x

**Published:** 2023-09-20

**Authors:** Mustafa Zakieh, Admas Alemu, Tina Henriksson, Nidhi Pareek, Pawan K. Singh, Aakash Chawade

**Affiliations:** 1 Department of Plant Breeding, Swedish University of Agricultural Sciences, 23422 Lomma, Sweden; 2 Lantmännen Lantbruk, Svalöv, Sweden; 3 Department of Microbiology, School of Life Sciences, Central University of Rajasthan, Bandarsindri, Kishangarh, Ajmer, Rajasthan 305801 India; 4 International Maize and Wheat Improvement Center (CIMMYT), Texcoco, Mexico

**Keywords:** Plant sciences, Plant genetics

## Abstract

Septoria tritici blotch (STB) is a destructive foliar diseases threatening wheat grain yield. Wheat breeding for STB disease resistance has been identified as the most sustainable and environment-friendly approach. In this work, a panel of 316 winter wheat breeding lines from a commercial breeding program were evaluated for STB resistance at the seedling stage under controlled conditions followed by genome-wide association study (GWAS) and genomic prediction (GP). The study revealed a significant genotypic variation for STB seedling resistance, while disease severity scores exhibited a normal frequency distribution. Moreover, we calculated a broad-sense heritability of 0.62 for the trait. Nine single- and multi-locus GWAS models identified 24 marker-trait associations grouped into 20 quantitative trait loci (QTLs) for STB seedling-stage resistance. The seven QTLs located on chromosomes 1B, 2A, 2B, 5B (two), 7A, and 7D are reported for the first time and could potentially be novel. The GP cross-validation analysis in the RR-BLUP model estimated the genomic-estimated breeding values (GEBVs) of STB resistance with a prediction accuracy of 0.49. Meanwhile, the GWAS assisted wRR-BLUP model improved the accuracy to 0.58. The identified QTLs can be used for marker-assisted backcrossing against STB in winter wheat. Moreover, the higher prediction accuracy recorded from the GWAS-assisted GP analysis implies its power to successfully select superior candidate lines based on their GEBVs for STB resistance.

## Introduction

Hexaploid winter wheat (*Triticum aestivum,* 2n = 6×  = 42, AABBDD) occupies the largest arable land in Northwest Europe^1^. An estimated 21% of world’s wheat production is lost due to diseases caused by pests and pathogens^2^. Septoria tritici blotch (STB) caused by the fungal species *Zymoseptoria tritici* (teleomorph *Mycosphaerella graminicola*)^3^ is the second leading disease for yield loss after stripe rust in Northwest Europe^4^. The disease causes an estimated yield loss of 5.51% in Northwest Europe that is two folds higher than its global average with 2.44%^2^. Yield losses up to 50% have been reported in epidemic years of the STB disease^5,6^. Most applied management strategies may still be as effective to overcome the disease outbreaks where wheat fields are subject to STB epidemics as a result of the airborne *Z. tritici* ascospores^7^. Moreover, STB management practices negatively affect the environment due to the intensive application of fungicides to control the disease accounting 70% of all fungicides used in wheat fungal disease management^8^. Besides, fungicide application costs up to $1.2 billion USD annually to manage the disease incidences in Europe^9^.

An integrated approach consisting of breeding for host resistance combined with other management practices is a sustainable strategy to mitigate STB impact. Wheat resistance breeding plays a major role in developing varieties with enhanced resistance lowering the environmental impact of fungicides application. So far, 22 qualitatively inherited major genes (*Stb* genes) have been identified for STB resistance in wheat^10,11^. However, the rapidly evolving *Z. tritici* populations as a result of sexual reproduction under field conditions caused the selection to be in favor of emerging new virulent strains that can overcome the identified major gene resistance^12^. In contrast, the higher number of minor to moderate effect minor genes inherited quantitatively are advantageous to that of major qualitatively inherited genes. Compared to qualitative resistance, quantitative resistance to STB is long acting against the range of diverse *Z. tritici* isolates. That is due to the cumulative effect of many genes adding minor to moderate resistance effects in non-isolate-specific resistance contrasting the short-lived and isolate–specific resistance triggered by major genes^10^.

To determine genetic resistance of hosts, phenotypic evaluation can be assessed from the development rate of disease symptoms on the leaf area. In other pathosystems, such as rusts, wheat genotypes conferring resistance to the disease show remarkably longer period for the appearance of infection symptoms (latency) where plants are able to slow down disease symptom development^13^*.* Similarly, STB latent phase (LP) from incubation to the appearance of disease symptoms can be used as an indicator for genotypic resistance evaluation under field and greenhouse conditions^14^. Under field conditions, factors such as sowing date, pycnidia concentration, environmental conditions and growth stage contribute to the length of STB latent period^15–17^. However, seedling-stage artificially inoculated plants under climate-controlled conditions overcome these factors to a large extent. In field conditions, the lower leaves tend to be more prone to STB as compared to younger ones^18^ and except for airborne conidia from neighboring fields, the lower leaves are the primary source for fresh inoculum production under conducive conditions. Hence, investigating genotypes with delayed symptom appearance of STB on seedling stage by exhibiting slower development of symptoms (prolonged or extended latent phase) is of great benefit for restricting STB infection spread. The mechanisms for such reduction of disease spread could be explained by the association of prolonged pre-symptomatic LP with reduced blotch size that is observed on infected wheat leaves^16^. By limiting size of the blotch, the capacity for producing larger amounts of the pathogen is restricted similar to other pathosystems^16,19^. Additionally, the delay in developing the necrotic phase during early growth stages of the plant renders the polycyclic nature of infection to be less efficient towards infecting new upper leaves. Genotypes with extended LP at seedling stage can express the quantitative seedling-stage resistance^20^.

The quantitatively inherited QTLs can be either seedling stage, adult-stage or all growth stage resistant to STB pathogen^21^. Linkage-based QTL mapping and genome-wide association studies have been instrumental to identify QTLs linked to either seedling-, adult- or all-stage STB resistance^10,22–24^. Several marker-trait associations have been detected via GWAS for STB resistance in wheat^25–31^.

The vast number of involved QTLs across the entire genome of wheat makes application of marker-assisted selection (MAS) for STB resistance a challenging task. Hence, evaluating plant materials with their overall genetic merit towards resistance to the disease through genomic prediction models is a more efficient and feasible approach^32^. Genomic prediction models estimate the breeding values of individual wheat plants from their overall SNP markers information. Regression models are trained with individuals called training population having the SNP markers and phenotypic data information. These models are then used to predict the genomic estimated breeding values (GEBVs) of individuals called the breeding or candidate population based on marker profile^33,34^. Then, selection of individuals in the breeding population is done exclusively from their predicted genetic information without the need to test on field or controlled conditions for phenotypic evaluation. The accuracy and usefulness of GS can be hampered by elements including low genetic diversity, insufficient training population size, and complex trait-genotype interactions, making it less suitable in some breeding situations^35^.

The current study was performed with 316 advanced breeding lines to identify QTLs linked to seedling-stage resistance against STB via GWAS analysis with various single- and multi-locus models. Additionally, the study aimed to estimate GEBVs of breeding lines for STB seedling-stage resistance through genomic prediction cross-validation analysis. To achieve these objectives, winter wheat lines were artificially inoculated and screened for seedling stage STB resistance in controlled condition by evaluating the spatio-temporal progression of the disease until the necrotic lesion development. Genotypes were sequenced with 25 K SNP array to discover genome-wide SNP markers.

## Materials and methods

### Plant material and experimental design

A set of 316 winter wheat advanced breeding lines provided by Lantmännen, Svalöv, Sweden, were evaluated for their resistance for STB at seedling-stage under controlled growth conditions. Four known varieties having three levels of resistance against the applied STB isolates were included as checks on the current experiment. These checks were Julius (resistant), KWS Kerrin and Stigg (moderate resistant) and Nimbus (susceptible).

The experiment was conducted in a randomized augmented block design with two replicates. Each replicate included 15 blocks and a single block comprised 23 breeding lines and 4 checks. Randomization of genotypes was done using the *design.dau* function in *Agricolae* package^36^ using R environment^37^.

### Plant growth condition and inoculation

Winter wheat seeds were stratified for 48 h in dark conditions at 3 °C on wetted filter paper followed by germination for 24 h at room temperature. Six to eight healthy germinated seeds were transferred to 8 × 8 × 9 cm plastic pots filled with potting peat soil (Gröna linjen, SW HORTO AB, Sweden) and kept for 4 days followed by thinning to two seedlings per pot. One gram of KH_2_PO_4_ per block was applied to promote root development and enhance seedlings recovery after thinning. The plants continued growing at 23/22 °C with 16/8 h of day/night cycle with relative atmospheric humidity (RH) of 60%. Plants were weekly fertilized with nitrogen and potassium fertilizer (SW-BOUYANT 7-1-5+MIKRO) added with equal amounts to individual blocks.

The inoculum was prepared by growing two single spore isolates collected from Alnarp and Svalöv, Sweden, following the protocol described by^18^. The inoculum concentration was adjusted to 10 × 10^5^ conidial spores/ml followed by adding the surfactant Tween^®^20 with 0.002% v/v. Three-leaf-stage 19-days-old winter wheat seedlings were spray-inoculated three times after marking the second and the third leaves. The leaves were left to dry after each spray for 20–30 min. On the third time spraying, plants were moved into a high humidity chamber with 90% RH at 23 °C for 48 h (16 and 8 h of light and dark conditions). After incubation, RH was lowered to 65% and continued until completion of the experiment.

In a small separate test, the virulence of the two isolates used in this study was initially examined by inoculating the four varieties with known resistance background to STB at seedling-stage including Kranich and Julius (resistant), Stigg (moderately resistant), and Nimbus (susceptible). Previous studies have identified cultivar Stigg as resistant to STB in different field conditions by extended latent phase before switching to the necrotrophic phase^14,38,39^. Odilbekov et al.^18^ identified cultivars Kranich and Nimbus as STB resistant and susceptible, respectively. Other studies showed that the cultivar Julius has high level of resistance to several diseases of wheat including STB^28,40^. For this purpose, 25 advanced breeding lines and 41 official trial lines were tested in an unreplicated augmented block design. The four cultivars (Kranich, Julius, Stigg and Nimbus) were used as checks dispersed across the 66 genotypes and replicated for a total of 19 times.

### STB disease evaluation

**Figure 1:**
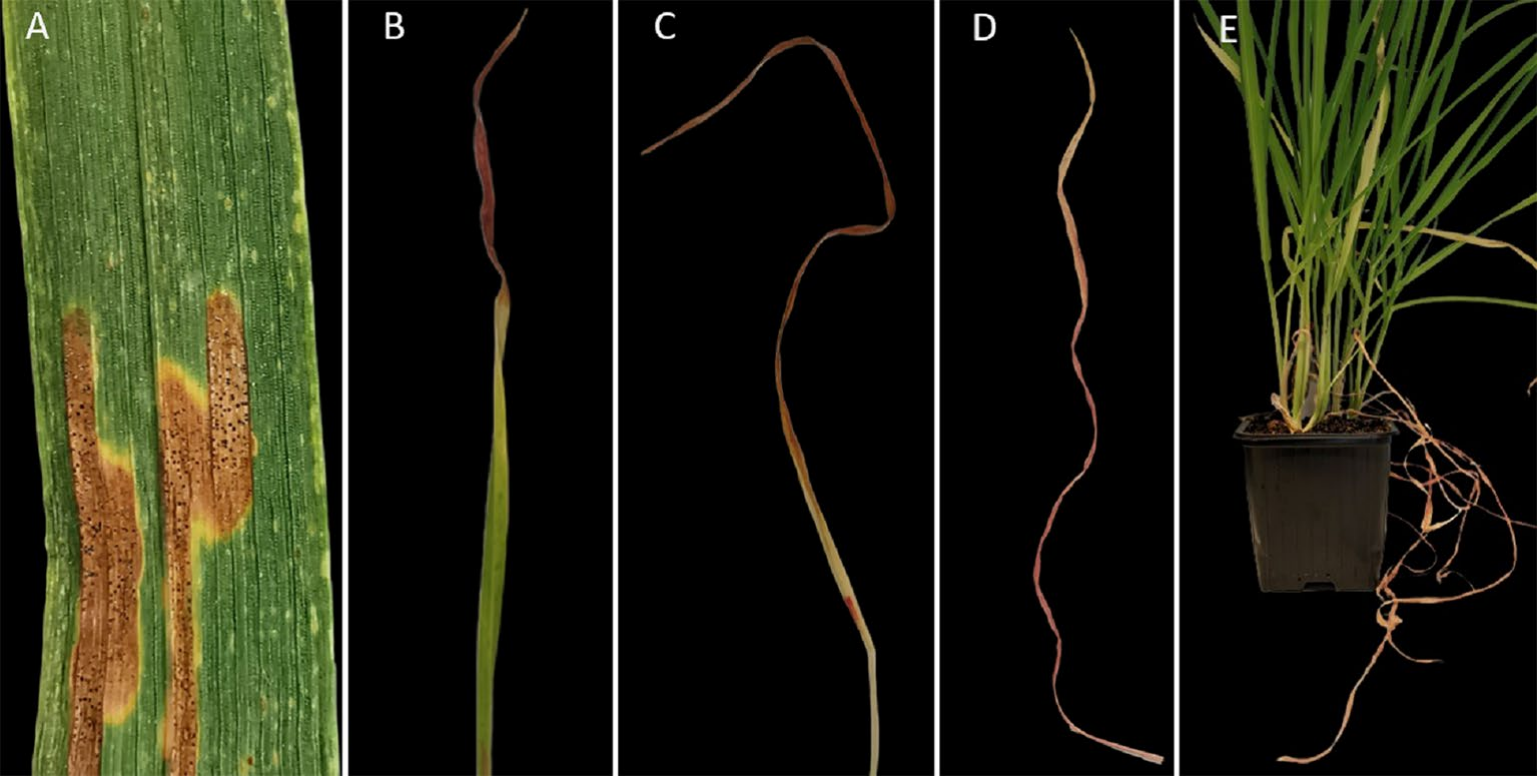
The Septoria tritici blotch (STB) disease lesions filled with pycnidia on infected winter wheat leaves under natural conditions (**A**), compared to STB symptoms observed in greenhouse artificially inoculated plants (**B**–**E**). Evaluating STB resistance in greenhouse-inoculated plants was carried out by scoring reddening necrotic leaf area that may be demonstrated partially (**B**) and (**C**) or fully (**D**) on the leaf. Scoring seedling stage infected leaves was done from the second and third leaves 15 days post inoculation (DPI), where they become the lower infected leaves (**E**).

Unlike natural infection (Fig. 1A), plants in greenhouse condition starts with general chlorosis that continues to spread across the whole leaf or partially from the leaf tip (Fig. 1B). Subsequently, reddish necrosis develops in place of chlorosis leading to collapse of the tissues in the infected area (Fig. 1C–E).

In the current study, the visual assessment of the disease was carried out 15 days post inoculation (DPI) on the second and third leaves of plants at seedling-stage under greenhouse conditions. Genotypes reaction to the disease was visually assessed every third day for four consecutive time points. An adjusted visual scaling scheme for disease severity was followed where reddish necrotic areas were estimated as percentage of disease severity ratio relative to the total leaf area following the procedure recently applied by Odilbekov et al.^18^.

### Phenotypic analysis

The phenotypic data analysis from the STB disease scoring was conducted in two steps. First, the four checks repeated in each block were used to adjust the means recorded from four consecutive scoring time points within each replicate using the *Agricolae* package^41^ following the model:

$${\text{Y}}_{{{\text{ij}}}} = {\text{u}} + {\text{G}}_{{{\text{ij}}}} + {\text{B}}_{{\text{j}}} +\upvarepsilon _{{{\text{ij}}}}$$

where Y_ij_ is the adjusted mean of the ith genotype in the jth block, u is the overall mean, G_ij_ is the effect of the ith genotype in the jth block, B_j_ is the effect form _j_th block and ɛ_ij_ the overall residual. An area under diseases progression curve (AUDPC) was estimated from the adjusted means using the following model:

$$\mathrm{AUDPC}= \sum_{\mathrm{i}=1}^{\mathrm{n}-1} \frac{\mathrm{Yi}+\mathrm{Y}(\mathrm{i}+1)}{2} \mathrm{X} (\mathrm{t}(\mathrm{i}+1)-\mathrm{t}{\text{i}})$$

where y_i_ = disease score at time t_i_; t_(i+1)_ − t_i_ = time (days) between two STB scoring time points; n = total number of scoring time points.

The second step was estimating the best linear unbiased prediction (BLUPs) across the two replicates from the adjusted mean AUDPC values of genotypes using META-R 6.04^42^ following the model:

$${\text{Y}}_{{{\text{ik}}}} = {\text{u}} + {\text{G}}_{{{\text{ik}}}} + {\text{R}}_{{\text{K}}} +\upvarepsilon _{{{\text{ik}}}}$$

where Y_ik_ is the BLUPs of ith genotype in the kth replicate, u is the overall mean, G_ik_ is the ith genotype effect in the kth replicate, R_K_ is the effect of Kth replicate and ɛ_ik_ the overall residual. Analysis of variance (ANOVA) and broad-sense heritability (*H*^2^) was retrieved in this step along with the adjusted mean values of AUDPC scores. Frequency distribution of AUDPC BLUPs was performed in the Minitab software package (Version 18).

### Genome-wide association analysis

The current winter wheat panel was previously genotyped using 25K SNP array by TraitGenetics GmbH, Germany that produced 24,145 SNP markers^43^. Finally, 10,120 SNP markers were selected for the GWAS analysis after excluding markers with minor allele frequency (MAF) ≤ 0.05 and with ≥ 0.2 missing values per individual. Nine GWAS models including 2 single and 7 multi-locus based models available in the Genome Association and Integrated Prediction Tool (GAPIT) 3.0^44^ and multi-locus random-SNP-effect mixed linear model (mrMLM) v4.0.2^45^ were employed to spot marker-trait associations. The fixed and random model circulating probability unification (FarmCPU)^46^, Bayesian-information and linkage-disequilibrium iteratively nested keyway (BLINK)^47^, multi-locus random SNP-effect mixed linear model (mrMLM)^48^, fast multi-locus random-SNP-effect efficient mixed model association (FASTmrEMMA)^49^, FAST multi-locus random-SNP-effect Mixed Linear Model (FASTmrMLM)^50^, polygene-background-control-based least angle regression plus empirical Bayes (pLARmEB)^51^ and integration of Kruskal–Wallis test with empirical Bayes under polygenic background control (pKWmEB)^52^ were the seven multi-locus models applied in the current analysis. Settlement of MLM under progressively exclusive relationship (SUPER)^53^ and general linear model (GLM)^54^ were the two single-locus models included in the GWAS analysis. Population structure as principal components (PCs) and pair-wise kinship similarity matrix were included with some of the statistical models required to overcome the false-positive marker-trait associations.

The Quantile–quantile (Q–Q) plots generated from the observed against expected − log_10_^*p*-values^ were used to evaluate the performance of the statistical models. Furthermore, the Bonferroni corrected threshold, applied to report major-QTLs, was calculated as, Bonferroni threshold =  − log_10_^(0.5/*n*)^, where *n* = number of total SNP markers applied to explore markers linked to STB resistance. Hence, Bonferroni threshold =  − log_10_^(0.5/*10,120*)^ = 4.3. Several previous GWAS studied with Bonferroni corrected threshold (*P* ≤ 0.05) have reported as major QTLs^20,55,56^.

Depending on the model, either − log_10_^*p*-value^ ≥ 4 (*P* ≤ 0.0001) or the logarithm of odds (LOD) scoring ≥ 4 were used as the exploratory significance thresholds to report the nominal-QTLs identified from the current marker-trait associations. Manhattan plots were generated for figurative visualization of the associated SNP markers across the wheat chromosomes. The SNP markers genetic positions were retrieved from the 90K SNPs consensus map^57^ while their physical position from the International Wheat Genome Sequence Consortium (IWGSC) v1.1^58^ and markers within 5 cM were considered as a single QTL.

### Genomic prediction analysis

The genomic prediction analysis was conducted with the ridge regression BLUP (RR-BLUP) model using the “rrBLUP” package^59^ in R environment following the mixed model formula:

$${\text{y}} = {\text{x}}\upbeta + {\text{Z}}\upmu +\upvarepsilon$$

where y is the vector of adjusted AUDPC mean score for STB resistance; x and β represents the designed matrix and vector of fixed effects, respectively; Z is the designed matrix for random effect SNP markers and μ is a vector of estimated random effect with μ ~ N(0, Gσ^2^μ), where G is a genomic relationship matrix calculated from all SNPs; and ε is the residual error.

The weighted RR-BLUP (wRR-BLUP) model was tested after fitting the five topmost significant GWAS-SNP markers (based on their* P* values) and fitted as fixed effects^60^. To avoid model overfitting, GWAS-SNPs were discovered from the training population (four-folds) excluding the validation population (one-fold) following the five-fold cross-validation scheme using FarmCPU model. The random sampling of genotypes into folds was done using the *sample()* function in R environment.

The prediction accuracy of models was assessed through cross-validation analysis. For the RR-BLUP model, individual genotypes were randomly split into training and validation sets with 80 and 20% ratio, respectively, and repeated for 500 iterations. However, in the wRR-BLUP model, the panel was first randomly divided in to five folds followed by GWAS analysis with only the four folds, which later used as training set. Then, the five topmost significant SNPs were identified and fitted as fixed effects in the prediction model. The genomic estimated breeding values of the remaining one fold was estimated with the trained model. The GWAS followed by genomic prediction analysis was repeated for 20 times.

Predictive ability was estimated as the correlation coefficient between the observed AUDPC adjusted mean values of genotypes and genomic estimated breeding values predicted for the test set based on the effect estimates of genotypes in the training set. Prediction accuracy was then calculated from prediction ability divided by the square root of traits’ broad sense heritability^61,62^.

### Ethics approval

All plant experiments were conducted according to relevant institutional, national, and international guidelines and legislation.

## Results

### Phenotypic analysis for STB resistance

**Figure 2:**
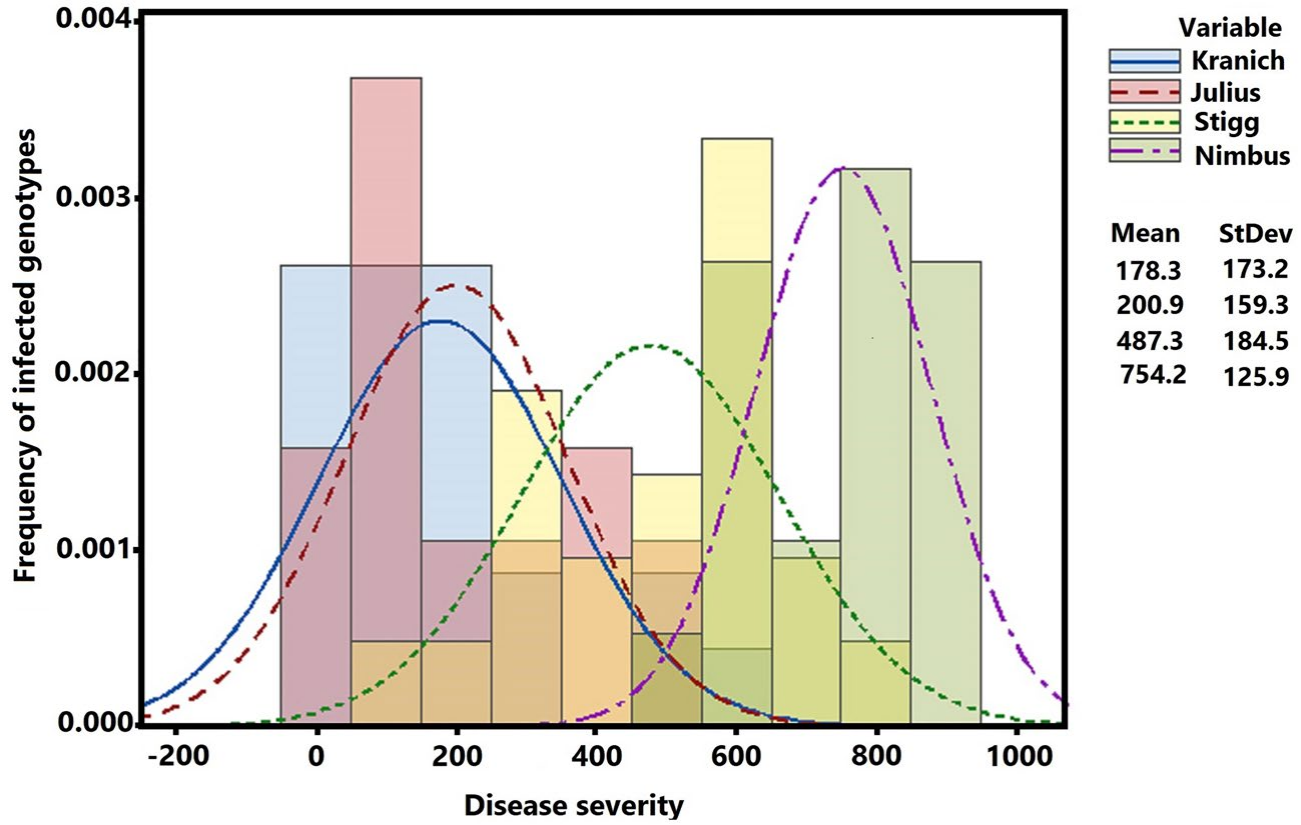
Frequency distribution for the area under diseases progression curve (AUDPC) score with the four winter wheat varieties having three known levels of resistance with the two applied *Zymoseptoria tritici* isolates.

The four known varieties, Kranich, Julius, Stigg and Nimbus having a varied degree of STB resistance was initially tested for any possible genotype-isolate/strain specific resistance via inoculating with the two isolates. The inoculation with the two mixed isolates elicited STB responses corresponding to the degree of resistance relative to their reported resistance backgrounds (Fig. 2). The four cultivars performed differently according to their resistance to the pathogen with an AUDPC score from below 10–900 for the most resistant and susceptible genotypes, respectively. The resistant cultivars Kranich and Julius scored low AUDPC values with means of 178.2 and 200.9, respectively, while the susceptible cultivar Nimbus had a mean AUDPC score of 754.2. Stigg exhibited moderate resistance with mean AUDPC score of 478.3 falling between the resistant and the susceptible cultivars (Fig. 2).

**Figure 3:**
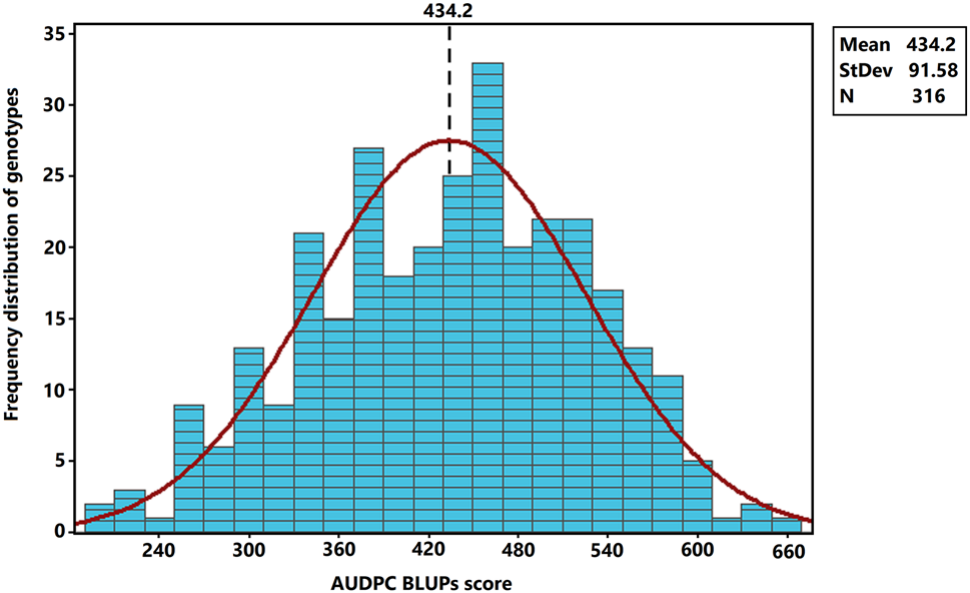
Distribution of the adjusted mean (BLUPs) AUDPC values of STB resistance recorded from the 316 winter wheat breeding lines. StDev, standard deviation; N, the number of tested genotypes.

Analysis of variance indicated a highly significant phenotypic differences among evaluated winter wheat lines for STB resistance (Supplementary Table S1). The AUDPC-BLUPs scores of the 316 breeding lines followed a normal distribution (Fig. 3) ranging from 193.6 to 666.2 with average and standard deviation values of 434.2 and 91.58, respectively. High broad-sense heritability (0.62) was recorded from the evaluated breeding lines.

### GWAS results

**Figure 4:**
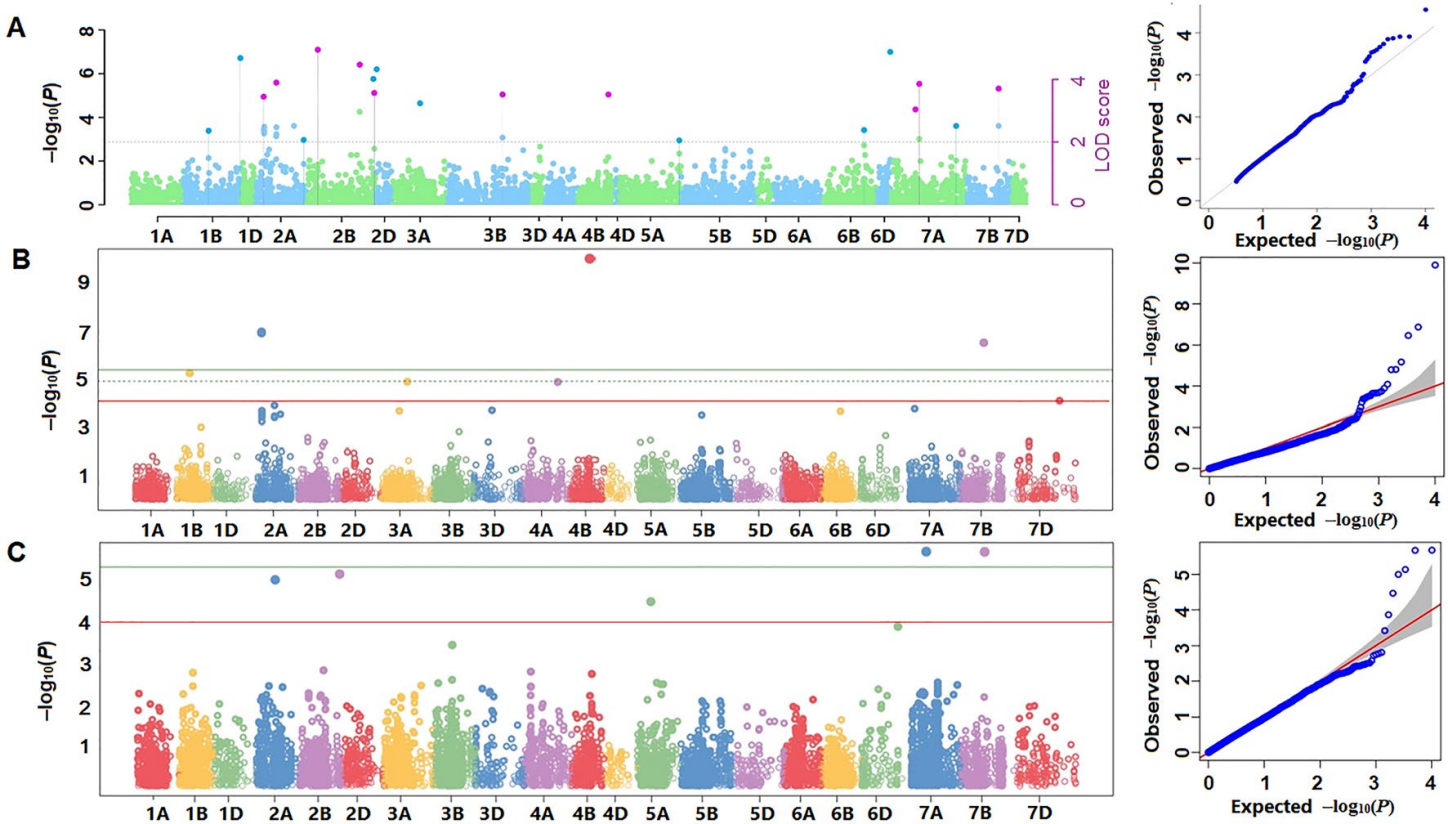
Manhattan (left) and Q-Q (right) plots of marker-trait associations identified for seedling-stage resistance to STB. (**A**) Manhattan (left) and Q-Q plots generated from the three multi-locus models (mrMLM, FASTmrMLM and FASTmrEMMA) with pink dots represents the SNP markers discovered by more than one model while dark blue dots represent the markers discovered by a single model. The dashed horizontal line on the diagram represents LOD score of 2.0. The other plots are for FarmCPU (**B**) and Blink (**C**) with green solid line representing the Bonferroni corrected thresholds at *P* = 0.05. The red solid line and green dash line represent the exploratory and false-discovery (FDR)-based thresholds at *P* = 0.0001 and 0.05, respectively.

The multi-locus GWAS models discovered 24 marker-trait associations (MTA) significantly linked to STB resistance (Supplementary Table S2). From these MTAs, 14 were detected with − logP ≥ 4 (*P* ≤ 0.0001) while the remaining 10 with − logP ≥ 3 (*P* ≤ 0.001) with atleast one tested model. These markers were located within 20 different QTLs identified on 14 chromosomes including 1B, 2A, 2B, 2D, 3A, 3B, 4A, 4B, 5A, 5B, 6D, 7A, 7B and 7D (Table 1). The AUDPC_BLUP distribution of genotypes based on the alleles of nine significantly linked markers with MAF > 0.35 is given in Supplementary Fig. 1. The SNP marker *AX-158596603*, under the major-QTL *SLUSTB_4*, on chromosome 2A (64 cM) had a highly significant association (*P* < 0.00001) and LOD score > 5 for STB resistance. Several models including Blink, pLARmEB and FASTmrEMMA identified this SNP marker highly significantly linked to host resistance to the pathogen. The other nearby SNP marker *AX-158573239* (63.8 cM) also exhibited a multi-model based highly significant association with the trait. The *SLUSTB_7* comprising two co-localized SNPs *Kukri_c17_1246* and *BS00083329_51* (135.5 cM) on chromosome 2B was the other major-QTL with a highly significant multi-model based association with STB resistance. The SNP marker *wsnp_Ex_c12220_19528388* (*SLUSTB_10*) on chromosome 3B (60.5 cM) has shown a highly significant association with the trait via several models including GLM, SUPER, BLINK, mrMLM, pLARmEB and pKWmEB (Table 1). The *SLUSTB_12* QTL comprised the marker *wsnp_CAP12_c1101_569783* on chromosome 4B (66.4 cM) which was detected with several models such as FarmCPU, mrMLM and pKWmEB with a highly significant threshold *P* < 0.00001 (Fig. 4). Four different models including FarmCPU, Blink, FASTmrEMMA and FASTmrMLM identified the SNP marker *Kukri_c51101_3510* (*SLUSTB_19*) on chromosome 7B (79.8 cM) highly significantly (*P* < 0.00001) linked to STB resistance. Four multi-locus models, pLARmEB, mrMLM, pKWmEB and FASTmrMLM identified the SNP *AX-158537280* (*SLUSTB_17)* on chromosome 7A (51.2 cM) significantly linked to STB resistance with the current panel. Moreover, several QTLs were identified with either at least with two multi-locus or both with the single- and multi-locus models. For instance, the SNPs on QTL *SLUSTB_5* (*Tdurum_contig54634_815,* 51.9 cM) and *SLUSTB_6* (*AX_158557660*, 81.4 cM) on chromosome 2B were detected by pLARmEB and pKWmEB models with a high significance threshold.

**Table 1:** List of QTLs, SNP markers and their effect, and models applied for the current GWAS analysis.

QTL	SNP	Chr	Pos (cM)	MAF	QTN effect		LOD score		Model
*SLUSTB_1*	*AX-89326139*	1B	40.3	0.21	NA	NA		FarmCPU***	
*SLUSTB_2*	*BS00066305_51*	1B	110.1	0.3	14.9	4.68		pKWmEB***	
*SLUSTB_3*	*AX-158572447*	2A	20.1	0.46	NA	NA		FarmCPU***	
*SLUSTB_4*	*AX-158573239*	2A	63.8	0.44	NA	NA		Blink***, FarmCPU*, GLM*	
	*AX-158596603*	2A	64	0.43	14.1–43.4	5.43		Blink**, pLARmEB*, FASTmrEMMA***	
*SLUSTB_5*	*Tdurum_contig54634_815*	2B	31.2	0.40	− 17.7 to − 16.0	4.51		pLARmEB***, pKWmEB***	
*SLUSTB_6*	*AX_158557660*	2B	81.4	0.19	17.8	5.21		pLARmEB***, pKWmEB***	
*SLUSTB_7*	*BS00083329_51*	2B	135.5	0.37	15	4.01		pKWmEB**	
	*Kukri_c17_1246*	2B	135.5	0.23	20.5–34	4.22		Blink***, FASTmrEMMA*, FASTmrMLM**	
*SLUSTB_8*	*AX-111036153*	2D	2.6	0.37	14.9	4.32		pLARmEB***	
*SLUSTB_9*	*wsnp_Ex_c8517_14315660*	3A	82.4	0.11	NA	NA		FarmCPU**	
*SLUSTB_10*	*wsnp_Ex_c12220_19528388*	3B	60.5	0.12	− 18.4	4.98		SUPER***, GLM**, Blink*, pLARmEB**, pKWmEB***	
*SLUSTB_11*	*Excalibur_c4325_1150*	4A	120.4	0.19	NA	NA		FarmCPU**	
*SLUSTB_12*	*wsnp_CAP12_c1101_569783*	4B	66.4	0.45	− 20.3	4.81		FarmCPU***, mrMLM**, pKWmEB***	
*SLUSTB_13*	*AX-158558835*	5A	44.7	0.48	NA	NA		Blink**	
*SLUSTB_14*	*AX-158585919*	5B	73.6	0.25	NA	NA		GLM**, SUPER*	
	*AX-158534098*	5B	73.6	0.25	NA	NA		GLM**, SUPER*	
*SLUSTB_15*	*IACX7841*	5B	134.4	0.25	NA	NA		GLM**, SUPER*	
	*wsnp_RFL_Contig3238_3265410*	5B	134.4	0.24	NA	NA		GLM**, SUPER*	
*SLUSTB_16*	*IAAV64*	6D	121.5	0.08	58.6	4.87		mrMLM***	
*SLUSTB_17*	*AX-158537280*	7A	51.2	0.28	-11.7	4.53		pLARmEB*, mrMLM***, pKWmEB**, FASTmrMLM***	
*SLUSTB_18*	*AX-108934671*	7B	28.1	0.42	NA	NA		SUPER**	
*SLUSTB_19*	*Kukri_c51101_351*	7B	79.8	0.04	35.4	5.66		FarmCPU***, Blink***, FASTmrEMMA*, FASTmrMLM**	
*SLUSTB_20*	*IAAV4542*	7D	142	0.13	NA	NA		FarmCPU**	

*Chr* chromosome, *MAF* minor allele frequency, *QTN* quantitative trait nucleotide, *LOD* logarithms of odds; *markers detected by the model(s) at *P* ≤ 0.001; ** markers detected by the model(s) at *P* ≤ 0.0001; *** markers detected by the model at *P* ≤ 0.00001; *NA* not applicable. QTN effect and LOD scores are estimated only in the multi-locus based mrMLM models.

### Genomic prediction analysis

**Figure 5:**
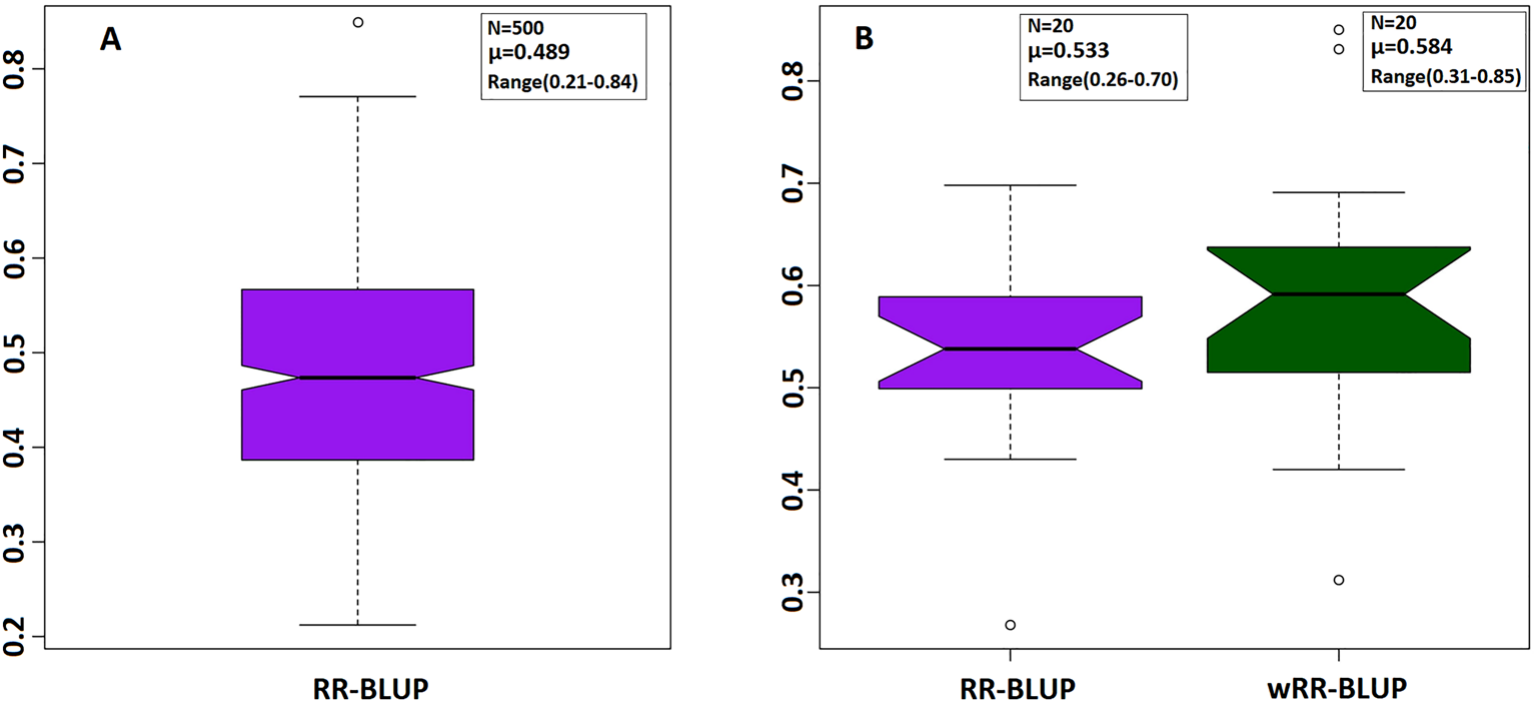
Genomic prediction for STB resistance using 316 winter wheat genotypes with two different statistical models. (**A**) Genomic prediction analysis with 80–20% training-validation sets cross-validation analysis conducted for 500 iterations with the RR-BLUP model. (**B**) The wRR-BLUP model with the five top-most significantly linked SNPs fitted as fixed effects. GWAS was conducted only in the training population and analysis was done for 20 times. Similarly, the 20 training-validation set splits were tested with the RR-BLUP model after excluding the fixed effect SNPs. N, number of iterations; μ, average values of iterations.

The RR-BLUP model with 80 and 20% of genotypes as training and validation sets, respectively, estimated the GEBVs for STB resistance with a prediction accuracy of 0.49 averaged from 500 iterations. The prediction accuracy with this model ranged from 0.21 to 0.84 (Fig. 5A). Genomic prediction of STB resistance with the current panel was further evaluated with the wRR-BLUP model. In this model, the five top-most significantly linked SNP markers identified only from the training set were fitted as fixed effects. Following the five-fold cross-validation scheme, GWAS was conducted with the training set (four-folds) with FarmCPU model masking a fold that was used as validation set and analysis was repeated for 20 times. The five-top-most GWAS-identified SNP markers can be find in the supplementary file (Supplementary Table S3).The wRR-BLUP model estimated the GEBVs of validation individuals for STB with a mean genomic prediction accuracy of 0.58 averaged from 20 runs (Fig. 5B). Similarly, the 20 training-validation set iterations were conducted with the RR-BLUP model excluding the five-fixed effect SNPs and the average prediction accuracy was 0.53. The genomic prediction accuracy of the wRR-BLUP and RR-BLUP models with the 20 iterations was in a range of 0.31–0.85 and 0.26–0.70, respectively.

## Discussion

### Phenotypic characterization and GWAS analysis for STB

Genetic improvement of winter wheat for resistance against STB is an indispensable approach to minimize the disease impact on wheat grain yield. The deployment of breeding lines with a wide genetic base can accelerate the development of wheat varieties resistant to wheat diseases, while keeping other important traits, such as yield optimized to a particular environment. The potential source of variation observed in the current studied breeding lines enabled the identification of genetic factors underpinning resistance to STB in juvenile plants under controlled conditions. Selection at seedling-stage is an essential step before testing in multi-environment trials hence an increased number of seedling-stage markers has been spotted that overlaps with known APR genes to STB resistance. This method minimizes the sheer number of susceptible individual lines from being tested in field conditions saving high amount of resources while increasing the selection accuracy.

Previous studies reported Stigg as a resistant variety for STB. However, in the current study, the variety fell below the most resistant variety Kranich and followed by Julius, while Nimbus was susceptible. Variations in seedling-stage resistance attributed to the spatio-temporal appearance in the latent phase leading to necrotic symptoms can aid in explaining the quantitative nature of resistance to STB of the tested genotypes. By using an AUDPC approach in association with the genomic markers of the tested genotypes, seedling-stage markers were detected that can potentially serve as a source of resistant novel QTLs. Additionally, the analysis showed a number of identified seedling-stage markers that co-localized with QTL identified as either seedling or adult-stage resistance.

In this study, 14 SNPs identified as significantly linked to STB- seedling-stage resistance were from six chromosomes located on the B sub-genome while the other 7 and 3 SNPs were on the chromosomes from the A and D sub-genomes, respectively. Several studies have similarly reported the B and A sub-genomes with highest number of STB major resistance genes where 16 out of 21 *Stb* genes were also identified from these two sub-genomes^10^.

The current GWAS analysis discovered twenty STB resistance QTLs out of which seven could potentially be novel. The identified novel markers were located on seven chromosomes including 1B, 2A, 2B, 5B (two), 7A, and 7D. The remaining 13 QTLs overlapped with previous reports either as seedling-stage (three), adult-stage (four) or all plant stage (six) QTLs for STB resistance. The majority of the identified MTAs were discovered from the multi-locus GWAS models even though few were exclusively discovered with single locus models such as the markers on 5B and 7B (Table 1). This could be due to the strong statistical power of the multi-locus models considering that they could manage detecting associations utilizing information from multiple markers simultaneously. Even though being able to detect lower number of MATs^63^, single-locus models are still an invaluable tool for marker discovery mainly for the discovery of markers with strong effect^64,65^ and can indicate to the robustness of the associations when MTAs are detected together with multi-locus models such as that on 3B (Table 1).

As to our knowledge, the seven identified QTLs in this study including *SLUSTB_2*, *SLUSTB_3*, *SLUSTB_6*, *SLUSTB_14*, and *SLUSTB_20* on chromosomes 1B, 2A, 2B, 7A and 7D, respectively, and *SLUSTB_14* & *SLUSTB_15* on chromosome 5B have not been reported in previous studies and could potentially be novel QTL sources to seedling-stage STB resistance in wheat.

Using the same isolates and under similar growth conditions, Odilbekov, et al.^18^ identified 10 MTAs associated with seedling-stage resistance located within 5 QTLs using plant materials comprised landraces and cultivars widely grown in the Nordic region for the last 100 years. Out of these, the two overlapped exactly with the currently identified QTLs. The marker *BS00066305_51* in *SLUSTB_2 QTL* was identified on the short arm of 1B distally located from the centromere at 110.1 cM. Generally, 1BS is a major source for *Stb* resistance genes including the *Stb2*^66^, and *Stb11*^10,67^. An earlier study has shown the presence of a QTL associated with several markers spanning a distance of 1.71 cM between 97.36 and 99.07 cM on 1BS identified from a panel of 175 winter wheat landraces and old cultivars at seedling-stage^18^. However, no previous QTL has been reported in close proximity to the current identified marker on the chromosome 1BS that leads us to report as a possible novel QTL for seedling-stage STB resistance in wheat. The *SLUSTB_3* associated with the marker *AX-158572447* at 20.1 cM on chromosome 2A appears as another potential novel marker associated with seedling-stage STB resistance. The marker *Tdurum_contig54634_815* from the *SLUSTB_5* QTL identified on the short arm of chromosome 2B (51.9 cM) was exactly overlapped with a previous report by Gerard et al. (2017) with a DArT marker *wPt2106* located at 51.86 cM. Another reported QTL on this chromosome region associated with adult-plant stage STB resistance was located on 65 cM^20^. On chromosome 2B, the marker *AX_158557660* (81.4 cM) from *SLUSTB_6* QTL could possibly be the other potential novel marker identified in the current study for seedling-stage STB resistance. Odilbekov, et al.^18^ identified a marker on chromosome 2B linked to seedling-stage STB resistance located at 96.99 cM, which is far from the currently detected marker by more than 15 cM. The *SLUSTB_14* and *SLUSTB_15* were the other two possibly novel QTL identified in the current analysis on chromosome 5B. The two significantly linked SNPs on *SLUSTB_14* (73.6 cM) identified in the current study were 13 cM far from the previously reported marker *Excalibur_c17489_804* identified with major effect QTL explaining 28% of the total phenotypic variation^23^. The markers *AX_158537280* on chromosomes 7A (51.2 cM) and *IAAV4542* on chromosome 7D on *SLUSTB_17* and *SLUSTB_20*, respectively, are the other two candidate novel QTLs spotted on the current study. Both *Stb4* and *Stb5* have been mapped on the short arm of 7DS chromosome arm^68,69^ while the currently identified marker *IAAV4542* was found on the long arm of chromosome 7DL located at 142 cM^70^. Therefore, it is not likely that this marker is associated with either of these genes.

The SNP marker *AX_89326139* (40.3 cM) on chromosome 1B appeared to overlap with the previously identified marker *IAAV3905* (41.3 cM) as APR QTL for STB^29^. Kidane et al.^71^ identified a QTL *qSTB.04* physically located at 587.28 Mbp identified in diverse panel of Ethiopian durum wheat landraces close to the currently detected marker *AX_89326139* at the physical distance of 544.5 Mbp. However, it is noteworthy to mention that these studies identified the respective markers on adult plants and the region could be a potential source of all-stage STB resistance in wheat. The two closely located markers *AX_158573239* (63.8 cM) and *AX_158596603* (64 cM) in *SLUSTB_4* overlapped with the recently identified QTL linked to seedling-stage STB resistance in a diverse germplasm of 185 genotypes associated with three *Z. tritici* isolates^72^. The two SNP markers of QTL *SLUSTB_7*, *BS00083329_51* and *Kukri_c17_1246* (135.5 cM) were only distant by 7.5 cM from the previously reported seedling-stage QTL *SRT_71-R3_2* on the long arm of chromosome 2B^73^. Similarly another nearby SNP marker *AX_94734086* (145 cM) was identified for APR to STB and claimed that this QTL could be part of the *Stb9* resistance gene involved in all stage STB resistance in wheat^29^.

The *SLUSTB_9* QTL with the SNP marker *wsnp_Ex_c8517_14315660* (82.4 cM) identified on chromosome arm 3AL was nearby to the marker *wsnp_Ex_c5929_10402147* (86.2 cM) previously spotted MTA for seedling-stage STB resistance^18^. Nearby to this QTL, a meta-QTL analysis for several biotic stresses reported four QTL situated between 80.4–87.1 cM^74^. This chromosome region has been identified as a source of several MTAs for STB resistance in previous investigations^71,75–77^. The QTL *SLUSTB_10* on chromosome 3B comprised the marker *wsnp_Ex_c12220_19528388* (60.5 cM) was detected by five different single- and multi-locus models. Other previous studies reported multiple MTAs for STB resistance with different *Z. tritici* isolate adjacent to this marker^72^. Alemu et al.^20^ reported a multi-environment stable MTA for APR to STB on chromosome 3B but far by 10 cM from the MTA identified in this study. These findings could lead us to speculate a potential QTL possibly existing on this chromosome region linked to all-stage STB resistance in wheat. Hence, further investigations on the validation of this candidate QTL region could enhance marker-assisted selection against the pathogen.

*Excalibur_c4325_1150* marker (*SLUSTB_11* QTL) was identified on chromosome arm 4AL located at 120.4 cM. Two nearby SNPs on the long arm of chromosome 4A, located on 121.4 cM and 122.5 cM, were previously reported significantly linked to STB resistance inoculated by a single *Z. tritici* isolate at seedling-stage^72^ and naturally infected winter wheat adult-plants^20^. Muqaddasi, et al.^29^ reported the SNP marker *wsnp_JD_c27162_22206547* that exactly overlapped with the currently identified marker on chromosome arm 4AL at 120.4 cM associated with STB APR detected from 371 artificially field-inoculated winter wheat genotypes.

It is yet unknown whether 4B chromosome is comprised major genes for STB resistance^10^. However, the currently identified marker *wsnp_CAP12_c1101_569783* (66.4 cM) on 4B had a highly significant association with STB resistance discovered by the multi-locus model FarmCPU with –log10 *P* > 9 (Fig. 4) and other two multi-locus models with LOD score > 4.8. Louriki et al.^73^reported two SNP markers located on the same chromosome arm identified from a panel of 377 advanced breeding lines of spring wheat associated with seedling-stage STB resistance each detected by one of the two tested isolates. The marker *RAC875_c24515_602* located at 76.8 cM^73^ could possibly from the same QTL with the marker identified in the current study. Using eight-founder MAGIC winter wheat population, Riaz et al. (2020) discovered several QTLs for adult-plant STB resistance on chromosome 4B including the marker *RAC875_c87897_333* (655.94 Mbp) which is found in nearby to the currently identified MTA. Hence, this region could be another source for QTLs of all-stage STB resistance.

*SLUSTB_8*, *SLUSTB_16* and *SLUSTB_20* were the three QTLs identified from the D sub-genome on chromosomes 2D, 6D and 7D, respectively. The SNP marker *AX_111036153* (2.6 cM) on chromosome 2D was identified in the current study that could be linked to a potential QTL identified for adult-plant STB resistance with strong association explaining a high portion of variation^23^. The marker *IAAV64* in the QTL *SLUSTB_16* discovered on chromosome 6D found at 466.55 Mbp. Similarly, Riaz et al.^23^ detected a very closely SNP marker *wsnp_Ex_c13188_20825019* (464.72 Mbp) linked to adult-stage STB resistance.

In general, 13 of the currently identified marker-trait associations have exactly overlapped with QTL regions previously reported by several studies. This could validate applied research protocols and procedures in the current study and increase the search and validation of valuable chromosome regions with resistance sources for the STB wheat pathogen. Furthermore, seven potentially novel QTLs identified in this study could also be used as a first brick in searching additional sources of resistance QTLs for the newly evolved pathogen strains.

### Genomic prediction for STB resistance

In addition to the well characterized *stb* genes, various minor- to major-effect QTLs are involved in host resistance against the STB pathogen in wheat^10^. Because of this, STB resistant wheat variety development through pyramiding of identified QTLs via marker-assisted selection has been a challenging task. Genomic prediction is a powerful method to accelerate the genetic gain of several quantitatively inherited traits in plant breeding^78^. Unlike the GWAS or linkage mapping methods, genomic prediction estimates the breeding values of individuals for traits of interest accounting all contributing QTLs based on their overall marker information^79^. This method is particularly an invaluable genomic tool for variety development with STB resistance and other similar traits controlled by several QTLs. The current study estimated the GEBVs of the 20% of 316 genotypes with two genomic prediction models trained with the remaining 80% of the panel. The RR-BLUP model estimated the GEBVs of STB resistance with prediction accuracy of 0.49 and 0.53 averaged from 500 and 20 iterations, respectively. A previous study reported a genomic prediction accuracy of 0.47 for STB resistance at seedling-stage with a 175 winter wheat panel comprising old cultivars and landraces^18^. With this panel, a low to moderate genomic prediction accuracy (0.15–0.35) was recorded for adult-stage STB resistance from multi-environmental field trials conducted in Denmark, Estonia, Lithuania, and Sweden^20^. Muqaddasi et al.^29^ reported a genomic prediction accuracy of 0.43 for adult-stage STB in 371 European winter wheat varieties. Juliana et al.^80^ reported a mean genomic prediction accuracy of 0.45 for STB adult plants resistance from CIMMYT International Bread Wheat Screening Nurseries (IBWSNs) with more than 600 lines evaluated at CIMMYT's research station, Toluca, Mexico for 3 years. However, several factors could affect the genomic prediction accuracy including the size of the training population, marker density, population structure, level of linkage disequilibrium and quality of the phenotypic data applied to train the model^78^. This RR-BLUP model assumes all markers share a common variance and similarly shrunk towards zero which leads to underestimation of major-effect QTLs^81^. To circumvent this drawback, the five top-most significantly linked SNPs identified via GWAS from training sets were fitted as fixed effects with the wRR-BLUP model. The model has shown an improved prediction accuracy by 5.1% compared to the RR-BLUP model tested in a similar training-validation population split. Previous studies reported beneficial genomic prediction accuracy improvements with the RR-BLUP model supplemented with QTL linked markers as fixed-effect^18,20,82^.

## Conclusion

The current study identified 24 marker-trait associations from nine single and multi-locus employed GWAS models. These marker-trait associations were found in 20 QTLs spread on 14 wheat chromosomes. We report seven as potentially novel QTLs spotted across seven chromosomes while the other 11 were overlapped with previously reported studies. The overlapped QTLs could help in the validation and application of marker-assisted backcrossing process while the newly spotted ones can assist on the searching of new resistant sources to the pathogen. Involving higher number of QTLs for STB resistance makes marker-assisted backcrossing a very challenging task. Hence, genomic prediction could play an immense role in accelerating the genetic gain of wheat breeding against the pathogen. The current genomic prediction analysis has shown a moderate to higher prediction accuracy to estimate the GEBVs of individual wheat genotypes for STB resistance. Furthermore, we report an improved prediction accuracy via QTL-assisted genomic prediction where the five topmost QTLs were used as fixed effect in the genomic prediction model.

### Supplementary Information

Supplementary Information 1.

Supplementary Information 2.

Supplementary Information 3.

## Data Availability

The original data contributions of the current study are included in the article/Supplementary Files.

## References

[1] Chawade A (2018). A transnational and holistic breeding approach is needed for sustainable wheat production in the Baltic Sea region. Physiol. Plantarum.

[2] Savary S (2019). The global burden of pathogens and pests on major food crops. Nat. Ecol. Evol..

[3] Quaedvlieg W (2011). Zymoseptoria gen. nov.: A new genus to accommodate Septoria-like species occurring on graminicolous hosts. Persoonia.

[4] Savary S (2019). The global burden of pathogens and pests on major food crops. Nat. Ecol. Evol..

[5] Ghaffary SMT, Chawade A, Singh PK (2018). Practical breeding strategies to improve resistance to Septoria tritici blotch of wheat. Euphytica.

[6] King JE, Jenkins JEL, Morgan WA (1983). The estimation of yield losses in wheat from severity of infection by Septoria species. Plant Pathol..

[7] Schuh W (1990). Influence of tillage systems on disease intensity and spatial pattern of Septoria leaf blotch. Phytopathology.

[8] Fones H, Gurr S (2015). The impact of Septoria tritici blotch disease on wheat: An EU perspective. Fungal Genet. Biol..

[9] Torriani SFF (2015). Zymoseptoria tritici: A major threat to wheat production, integrated approaches to control. Fungal Genet. Biol..

[10] Brown JKM, Chartrain L, Lasserre-Zuber P, Saintenac C (2015). Genetics of resistance to Zymoseptoria tritici and applications to wheat breeding. Fungal Genet. Biol..

[11] Yang N, McDonald MC, Solomon PS, Milgate AW (2018). Genetic mapping of Stb19, a new resistance gene to Zymoseptoria tritici in wheat. Theor. Appl. Genet..

[12] McDonald BA, Mundt CC (2016). How knowledge of pathogen population biology informs management of Septoria tritici blotch. Phytopathology.

[13] Broers LHM (1997). Components of quantitative resistance to yellow rust in ten spring bread wheat cultivars and their relations with field assessments. Euphytica.

[14] Hehir JG (2018). Temporal and spatial field evaluations highlight the importance of the presymptomatic phase in supporting strong partial resistance in Triticum aestivum against Zymoseptoria tritici. Plant Pathol..

[15] Henze M, Beyer M, Klink H, Verreet JA (2007). Characterizing meteorological scenarios favorable for Septoria tritici infections in wheat and estimation of latent periods. Plant Dis..

[16] Hess DE, Shaner G (1987). Effect of moisture and temperature on development of Septoria tritici blotch in wheat. Phytopathology.

[17] Shaw MW (1990). Effects of temperature, leaf wetness and cultivar on the latent period of mycosphaerella-graminicola on winter-wheat. Plant Pathol..

[18] Odilbekov F, Armoniené R, Koc A, Svensson J, Chawade A (2019). GWAS-assisted genomic prediction to predict resistance to Septoria tritici blotch in Nordic winter wheat at seedling stage. Front. Genet..

[19] Ramirez-Cabral NYZ, Kumar L, Shabani F (2017). Global risk levels for corn rusts (Puccinia sorghi and Puccinia polysora) under climate change projections. J. Phytopathol..

[20] Alemu A (2021). Genome-wide association analysis and genomic prediction for adult-plant resistance to Septoria tritici blotch and powdery mildew in winter wheat. Front. Genet..

[21] Yashavanthakumar KJ (2018). Phenotyping slow leaf rusting components and validation of adult plant resistance genes in exotic wheat germplasm. Australas. Plant Pathol..

[22] Naz AA, Klaus M, Pillen K, Léon J (2015). Genetic analysis and detection of new QTL alleles for Septoria tritici blotch resistance using two advanced backcross wheat populations. Plant Breed..

[23] Riaz A (2020). Genetic analysis using a multi-parent wheat population identifies novel sources of Septoria tritici blotch resistance. Genes-Basel.

[24] Tamburic-Ilincic L, Rosa SB (2019). QTL mapping of Fusarium head blight and Septoria tritici blotch in an elite hard red winter wheat population. Mol. Breed..

[25] Ando K (2018). Genome-wide associations for multiple pest resistances in a Northwestern United States elite spring wheat panel. PLoS ONE.

[26] Gerard GS, Borner A, Lohwasser U, Simon MR (2017). Genome-wide association mapping of genetic factors controlling Septoria tritici blotch resistance and their associations with plant height and heading date in wheat. Euphytica.

[27] Gurung S (2014). Genome-wide association study reveals novel quantitative trait loci associated with resistance to multiple leaf spot diseases of spring wheat. PLoS ONE.

[28] Kollers S (2013). Genetic architecture of resistance to Septoria tritici blotch (Mycosphaerella graminicola) in European winter wheat. Mol. Breed..

[29] Muqaddasi QH (2019). Genome-wide association mapping and prediction of adult stage Septoria tritici blotch infection in European winter wheat via high-density marker arrays. Plant Genome-US.

[30] Vagndorf N (2017). Genomewide association study reveals novel quantitative trait loci associated with resistance towards Septoria tritici blotch in North European winter wheat. Plant Breed..

[31] Yates S (2019). Precision phenotyping reveals novel loci for quantitative resistance to Septoria tritici blotch. Plant Phenomics.

[32] Alemu A, Batista L, Singh PK, Ceplitis A, Chawade A (2023). Haplotype-tagged SNPs improve genomic prediction accuracy for Fusarium head blight resistance and yield-related traits in wheat. Theor. Appl. Genet..

[33] Hastie T, Tibshirani R, Friedman J (2009). The Elements of Statistical Learning: Data Mining, Inference, and Prediction.

[34] Meuwissen TH (2009). Accuracy of breeding values of 'unrelated' individuals predicted by dense SNP genotyping. Genet. Sel. Evol..

[35] Juliana P (2022). Genomic selection for wheat blast in a diversity panel, breeding panel and full-sibs panel. Front. Plant Sci..

[36] De Mendiburu, F. Agricolae: Statistical procedures for agricultural research. *R package version* **1** (2014).

[37] *R: A language and environment for statistical computing* (R Foundation for Statistical Computing, 2022).

[38] Benbow HR (2020). Insights into the resistance of a synthetically-derived wheat to Septoria tritici blotch disease: Less is more. BMC Plant Biol..

[39] Brennan CJ (2020). Taxonomically restricted wheat genes interact with small secreted fungal proteins and enhance resistance to Septoria tritici blotch disease. Front. Plant Sci..

[40] Laidig F (2021). Breeding progress of disease resistance and impact of disease severity under natural infections in winter wheat variety trials. Theor. Appl. Genet..

[41] De Mendiburu F (2014). Agricolae: Statistical procedures for agricultural research. R package version.

[42] Alvarado, G. *et al.* META-R (Multi Environment Trail Analysis with R for Windows) Version 5.0. *CIMMYT Research Data & Software Repository Network* **23**, 2015 (2015).

[43] Zakieh M (2021). Characterizing winter wheat germplasm for fusarium head blight resistance under accelerated growth conditions. Front. Plant Sci..

[44] Lipka AE (2012). GAPIT: Genome association and prediction integrated tool. Bioinformatics.

[45] Zhang YW (2020). mrMLM v4.0.2: An R Platform for multi-locus genome-wide association studies. Genom Proteom. Bioinf..

[46] Liu X, Huang M, Fan B, Buckler ES, Zhang Z (2016). Iterative usage of fixed and random effect models for powerful and efficient genome-wide association studies (vol 12, e1005767, 2016). PLoS ONE.

[47] Huang M, Liu XL, Zhou Y, Summers RM, Zhang ZW (2019). BLINK: A package for the next level of genome-wide association studies with both individuals and markers in the millions. Gigascience.

[48] Wang SB (2016). Improving power and accuracy of genome-wide association studies via a multi-locus mixed linear model methodology. Sci. Rep.-UK.

[49] Wen YJ (2018). Methodological implementation of mixed linear models in multi-locus genome-wide association studies. Brief. Bioinform..

[50] Tamba, C. L. & Zhang, Y.-M. A fast mrMLM algorithm for multi-locus genome-wide association studies. *biorxiv*, 341784 (2018).

[51] Zhang J (2017). pLARmEB: Integration of least angle regression with empirical Bayes for multilocus genome-wide association studies. Heredity.

[52] Ren WL, Wen YJ, Dunwell JM, Zhang YM (2018). pKWmEB: Integration of Kruskal-Wallis test with empirical Bayes under polygenic background control for multi-locus genome-wide association study. Heredity.

[53] Wang QS, Tian F, Pan YC, Buckler ES, Zhang ZW (2014). A SUPER powerful method for Genome Wide Association Study. PLoS ONE.

[54] Pritchard JK, Stephens M, Donnelly P (2000). Inference of population structure using multilocus genotype data. Genetics.

[55] Maccaferri M (2016). Prioritizing quantitative trait loci for root system architecture in tetraploid wheat. J. Exp. Bot..

[56] Alemu A (2021). Genome-wide association analysis unveils novel QTLs for seminal root system architecture traits in Ethiopian durum wheat. BMC Genomics.

[57] Wang S (2014). Characterization of polyploid wheat genomic diversity using a high-density 90 000 single nucleotide polymorphism array. Plant Biotechnol. J..

[58] IWGSC. Shifting the limits in wheat research and breeding using a fully annotated reference genome. *Science* **361**, eaar7191, 10.1126/science.aar7191 (2018). 10.1126/science.aar7191 30115783

[59] Endelman JB (2011). Ridge regression and other kernels for genomic selection with R package rrBLUP. Plant Genome.

[60] Spindel JE (2016). Genome-wide prediction models that incorporate de novo GWAS are a powerful new tool for tropical rice improvement. Heredity.

[61] Legarra AS, Robert-Granié CL, Manfredi E, Elsen J-M (2008). Performance of genomic selection in mice. Genetics.

[62] Chen CY (2011). Genome-wide marker-assisted selection combining all pedigree phenotypic information with genotypic data in one step: An example using broiler chickens. J. Anim. Sci..

[63] Taub M, Schwender H, Younkin S, Louis T, Ruczinski I (2013). On multi-marker tests for association in case-control studies. Front. Genet..

[64] Jaiswal V (2016). Genome wide single locus single trait, multi-locus and multi-trait association mapping for some important agronomic traits in common wheat (*T. aestivum* L.). PLoS ONE.

[65] Xu Y (2018). Genome-wide association mapping of starch pasting properties in maize using single-locus and multi-locus models. Front. Plant Sci..

[66] Liu YY, Zhang LL, Thompson IA, Goodwin SB, Ohm HW (2013). Molecular mapping re-locates the Stb2 gene for resistance to Septoria tritici blotch derived from cultivar Veranopolis on wheat chromosome 1BS. Euphytica.

[67] Chartrain L (2005). Genetics of resistance to septoria tritici blotch in the Portuguese wheat breeding line TE 9111. Theor Appl Genet.

[68] Adhikari TB (2004). Molecular mapping of the Stb4 gene for resistance to Septoria tritici blotch in wheat. Phytopathology.

[69] Arraiano L, Worland A, Ellerbrook C, Brown J (2001). Chromosomal location of a gene for resistance to septoria tritici blotch (*Mycosphaerella graminicola*) in the hexaploid wheat’Synthetic 6x’. Theor Appl Genet.

[70] Yao E (2022). GrainGenes: A data-rich repository for small grains genetics and genomics. Database-Oxford.

[71] Kidane YG (2017). Genome-Wide Association Study of Septoria tritici Blotch resistance in Ethiopian durum wheat landraces. Front. Plant Sci..

[72] Mahboubi, M. *et al.* Genome-wide association mapping in wheat reveals novel QTLs and potential candidate genes involved in resistance to septoria tritici blotch (2021).

[73] Louriki S (2021). Identification of resistance sources and Genome-Wide Association mapping of Septoria tritici blotch resistance in spring bread wheat germplasm of ICARDA. Front. Plant Sci..

[74] Soriano JM, Colasuonno P, Marcotuli I, Gadaleta A (2021). Meta-QTL analysis and identification of candidate genes for quality, abiotic and biotic stress in durum wheat. Sci. Rep..

[75] Eriksen L, Borum F, Jahoor A (2003). Inheritance and localisation of resistance to *Mycosphaerella graminicola* causing Septoria tritici blotch and plant height in the wheat (*Triticum aestivum* L.) genome with DNA markers. Theor. Appl. Genet..

[76] Ghaffary SMT (2011). Genetic analysis of resistance to septoria tritici blotch in the French winter wheat cultivars Balance and Apache. Theor. Appl. Genet..

[77] Radecka-Janusik M, Czembor PC (2014). Genetic mapping of quantitative trait loci (QTL) for resistance to Septoria tritici blotch in a winter wheat cultivar Liwilla. Euphytica.

[78] Crossa J (2017). Genomic selection in plant breeding: Methods, models, and perspectives. Trends Plant Sci..

[79] Meuwissen THE, Hayes BJ, Goddard ME (2001). Prediction of total genetic value using genome-wide dense marker maps. Genetics.

[80] Juliana P (2017). Comparison of models and whole-genome profiling approaches for genomic-enabled prediction of Septoria tritici blotch, stagonospora nodorum blotch, and tan spot resistance in wheat. Plant Genome.

[81] Lorenz, A. J. *et al.* in *Advances in Agronomy* 77–123 (Elsevier, 2011).

[82] Gaikpa DS (2021). Exploiting genetic diversity in two European maize landraces for improving Gibberella ear rot resistance using genomic tools. Theor. Appl. Genet..

