# Supplementary material for: Exploring GWAS and genomic prediction to improve Septoria tritici blotch resistance in wheat
Source: Sci Rep. 2023 Sep 20;13:15651. doi: 10.1038/s41598-023-42856-x (PMC10511425; doi:10.1038/s41598-023-42856-x)
Supplement: Supplementary file 1 — Supplementary Information 1. [file 41598_2023_42856_MOESM1_ESM.docx]

**Supplementary figure 1**. Breeding distribution with the alleles for nine SNPs detected by GWAS models as marker trait associations with minor allele frequency threshold >0.35.

|  |  |
| --- | --- |
|  |  |
|  |  |
|  |  |
|  |  |
